# Screening, diagnosis, and long-term health outcomes in developing countries—The case of hypertension

**DOI:** 10.1371/journal.pone.0208466

**Published:** 2018-12-05

**Authors:** Younoh Kim, Vlad Radoias

**Affiliations:** Department of Economics and International Business, Sam Houston State University, Huntsville, Texas, United States of America; University of Essex, UNITED KINGDOM

## Abstract

Hypertension is a rapidly growing problem in developing countries. At the same time, due to its asymptomatic nature, the afflicted population is largely unaware of being hypertensive. Due to a lack of resources, routine medical exams are very rare in developing countries and many sick individuals remain undiagnosed. Using a large sample of hypertensive individuals from Indonesia, we show the importance of being diagnosed. Diagnosed individuals exhibit lower systolic and diastolic blood pressure, and overall lower probability of remaining hypertensive than undiagnosed individuals. We also show the main channels through which this is achieved: taking medication, routinely monitoring one’s blood pressure, and engaging in moderate physical activities. We also point to channels through which additional benefits could be realized, but that are currently ineffective: dietary changes and maintaining a healthy body weight. Combined, these results point to the importance of directing public policy towards addressing the under-diagnosis problem and educating the public of the benefits of adopting a healthy life-style.

## Introduction

Periodic health screening is a routine that most people living in developed economies have grown accustomed to. Medical professionals everywhere recommend routine screenings that can discover chronic diseases in their infancy and allow for adequate interventions to be formulated and prescribed. However, due to a multitude of socio-economic deficiencies, this practice is less common in underdeveloped or developing economies. Due to a general lack of resources, routine medical exams are very rare in developing countries. Furthermore, due to a historical struggle to fight infectious diseases, many health care providers and even governments in developing countries are underestimating the severity and prevalence of chronic diseases. This leads to extremely high rates of chronic diseases under-diagnosis, which can further lead to more serious health problems and ultimately increase mortality rates.

The problem is even more pronounced for asymptomatic health conditions, such as hypertension. Hypertension can go unnoticed for years, but can have major and even fatal consequences if left untreated. Elevated blood pressure is a major risk for coronary heart disease and can lead to stroke and organ damage while being either symptomatic or asymptomatic [1]. The World Health Organization (WHO) defines hypertension as having blood pressure of at least 140 mm Hg systolic/90 mm Hg diastolic. Most people who are hypertensive manifest absolutely no signs or symptoms, even at highly elevated levels. Therefore, in the absence of routine medical exams, hypertension is extremely hard to screen and diagnose, as people who suffer from it are largely unaware of its presence. As expected, developing countries have extremely high rates of hypertension under-diagnosis. Previous studies report rates of 49% under-diagnosis for men in China [2]. Others confirm this by finding that in Indonesia, 67% of the men and 54% of the women who were actually found hypertensive in a national survey had never been previously diagnosed by a doctor [3].

Under-diagnosis has been linked to a variety of possible causes like socio-economic indicators such as income and education, community characteristics and infrastructure, and even individual risk and time preferences [2] [3] [4]. From a health economics perspective, improving diagnosis rates can be therefore achieved through making investments in education, local infrastructure, and access to healthcare. These investments are costly and there is unfortunately not a lot of prior quantitative evidence regarding the benefits of early diagnosis. Furthermore, some of the prior evidence is actually not that encouraging. For instance [5] and [6] find no significant effects of health checks on total mortality. Cohen et al. [7] find an equally pessimistic result that prevention is not cheaper than treatment. Somewhat related, Burris et al. [8] present evidence from a systematic literature review and meta-analysis that more than half of patients diagnosed with lung or throat cancer continue to smoke. We urge researchers not to apply these results indiscriminately.

We argue that meaningful empirical studies addressing this issue should be more narrowly defined and more carefully constructed from a theoretical perspective. The theoretical benefits of screening and diagnosis are primarily informational and by itself diagnosis does not improve outcomes. But when treatments are available, the diagnosis information can further work towards improving one’s health through two distinct channels which can be loosely referred to as professionally directed treatment and self-directed treatment. By professionally directed treatment we mean to include things like medication, medical interventions, and things that generally require the assistance of a health-care professional. By self-directed treatment we mean to include things like adopting a better diet, quitting smoking and drinking, and other life-style changes that a health-care professional might recommend but that are ultimately personal choices that generally require a significant behavioral effort. When discussing the effect of diagnosis on health outcomes it is therefore important to evaluate the existence, efficacy, and cost for both types of treatments, which implies that different health conditions might present extremely different results. We might not observe the desirable effects when looking at mortality rates of a hard to cure condition or when considering the incidence of smoking, which is highly addictive and hard to quit. But we might observe such effects with other health issues that are easier to address.

For the case of hypertension, it is generally cheap to both screen and diagnose it and also to treat it or at least control it using a variety of available medication. Hypertensive patients are also recommended to maintain a normal body weight, engage in moderate physical activities, change their diets, and limit the intake of certain substances like alcohol or sodium [9]. However, these self-care methods might require higher behavioral costs that need to be incurred by each individual patient. We therefore expect hypertension diagnosis to have clear positive health consequences that are achieved mainly through the cheaper channels like medication and easy to implement life-style choices. Because these cheaper alternatives act like substitutes to the behaviorally more expensive life-style choices, we do not expect individuals to dramatically alter their diets in response to being diagnosed with hypertension.

Using recent data from Indonesia, we estimate significant benefits of screening and diagnosis on the prevalence and severity of hypertension. Hypertensive patients who are diagnosed are more likely to take medication, engage in routine blood pressure checks, quit smoking, and engage in moderate physical activities. While major life-style changes prompted by diagnosis are somewhat limited and do not include healthier dietary changes, diagnosis has a clear beneficial role on lowering both systolic and diastolic blood pressure, and ultimately reducing the incidence and severity of hypertension.

## Materials and methods

In order to estimate the effects of being previously diagnosed on later blood pressure, we use data from the Indonesia Family Life Survey (IFLS). IFLS is a large longitudinal survey spanning over 20 years and containing a representative sample of Indonesian households. IFLS collects a vast number of demographic and socio-economic indicators at the individual, household, and community level. For our study, we use data from the latest IFLS wave, which was fielded in 2015. The data used is fully anonymized, survey data, made publicly available by the Rand Corporation. IRB approval was obtained both at Rand and in Indonesia when the surveys were fielded.

As part of collecting health data, trained nurses took the blood pressure of all IFLS respondents 15 years or older. These blood pressure measurements were taken three times. We dropped the first measurement in order to alleviate measurement errors due to respondents’ nervousness, then took the average of the last two measurements to construct our systolic and diastolic blood pressure variables. Using these blood pressure measurements, we assembled our sample by including all respondents who according to the WHO definition can be classified as hypertensive. In addition to this, we added to the sample those respondents who were previously diagnosed as hypertensive and those respondents who were found taking hypertension medication at the time of the survey, even though their measured blood pressure would not qualify them as hypertensive. The reasoning is that medication or other types of treatment following diagnosis might bring blood pressure to normal levels, but these patients are still hypertensive. All the patients were asked if they had been previously diagnosed with hypertension by a medical professional.

Our sample contains 7585 hypertensive individuals between the ages of 15 and 103, out of which only 3242 were previously diagnosed with hypertension. That implies about 57% of the hypertensive individuals were not aware of their condition. This under-diagnosis rate figure is quite high but in line with previous findings from the literature. The raw IFLS sample contains more hypertensive respondents than reported here, but some contain missing information and cannot be used in our regression analysis. We only report here the full sample of individuals for which all the information is available and can be used in the regression analysis. Table 1 presents some important summary statistics.

**Table 1:** Summary statistics.

**Blood Pressure Measurements**	**Mean (St.Dev.)**	**Range**
Systolic	150.16 (21.98)	90.5–240.5
Diastolic	89.90 (12.18)	48–148.5
**Sample Composition**	**Respondents**	**Percentage out of Total**
Total Sample	7585	100
Respondents with High BP	6261	82.54
Respondents Previously Diagnosed	3242	42.74
Respondents with Normal BP	1324	17.46
Respondents Taking Medication	1118	14.74
**Socio-economic Caracteristics**	**Mean (St.Dev.)**	**Range**
Age	48.6 (15.95)	15–103
Years of Education	7.36 (5.02)	0–24
log PCE	13.74 (0.72)	11.19–18.53
**General Health Status**	**Respondents**	**Percentage out of Total**
Being Overweight (BMI≥25)	3362	44.32
Being in Poor GHS	2260	29.8

Our outcome variables will be the two blood pressure measures (systolic and diastolic) and an indicator variable that takes value 1 if the respondent is currently hypertensive according to the WHO definition (140 mm HG systolic/90 mm Hg diastolic), and 0 otherwise. While it may seem redundant to report both the hypertensive status and the systolic/diastolic blood pressures separately, these measures can tell different stories. Heart attack risks for instance are more closely related to the systolic numbers, and isolated systolic hypertension can be quite common. We then estimate the effect of having been previously diagnosed on these current measures of health status, while controlling for a series of individual specific socio-economic and health characteristics. For each of the aforementioned 3 blood pressure measures, we hence estimate the following equation:

$$\begin{array}{c} BPMeasure_{i}=\alpha +\beta \cdot Diagnosis_{i}+\gamma X_{i}+\varepsilon_{i} \end{array}$$

where *Diagnosis*_*i*_ is an indicator variable equal to 1 if respondent *i* has been diagnosed with hypertension in the past and 0 oherwise, *X*_*i*_ represents a vector of characteristics specific to respondent *i*, and *ε*_*i*_ is the error term that is assumed to be uncorrelated with the diagnosis status.

As control variables that are included in the vector *X*_*i*_, we use respondent’s age and age squared (to account to possible non-linearities with respect to age), respondent’s years of formal education, the log of per capita expenditures (logPCE) to proxy for household income, an indicator for being overweight, an indicator for poor general health status, and an indicator for sex. Bound and Krueger [10] show that there are systematic measurement errors in household income, and PCE has been used ever since in the literature as a proxy as it is less prone to such measurement errors. WHO defines being overweight as having a body mass index greater or equal to 25. We hence coded the overweight indicator equal to 1 if respondents have BMI of at least 25 and 0 for BMI lower than 25. For the general health status, respondents were asked to evaluate their health as being either very healthy, somewhat healthy, somewhat unhealthy, or unhealthy. We coded the Poor GHS indicator equal to 1 if respondents chose either somewhat unhealthy or unhealthy.

Note that some previous research shows an association between height and hypertension [11] and between height and income [12]. Both of these studies use Indonesian data from IFLS. Since income can potentially be an important channel for both diagnosis and disease management, the question arises if height should be added to our regression analysis as an additional control. We tried doing that and found no statistically significant impact of height and identical results for all other variables. This was expected since we already control for the income aspect through per capita expenditures, and height is already accounted for through BMI.

Estimating these equations will yield the association of a prior diagnosis with current health outcomes. However, as argued before, the diagnosis is merely informational and does not treat the affliction by itself. It needs to be followed by treatment. In order to see the channels through which potential health improvements are realized, we estimate the effect of a prior diagnosis on a series of potential courses of treatment and control. We want to see if a prior diagnosis leads to taking medication, increasing engagement in physical activity, changing ones dietary habits, reducing body weight, quitting smoking, etc. These equations are similar with the main equation involving the blood pressure measures and are explained in more detail in the results section.

## Results and discussion

Table 2 presents the estimated effects of having been previously diagnosed of hypertension on current hypertension. Current here refers to the time of the 2015 IFLS wave, and all the blood pressure measures used come from IFLS. We performed 3 different regressions, using the same set of explanatory variables, but three different dependent variables that speak to hypertension.

**Table 2:** The effects of diagnosis on hypertension.

Explanatory Variable	Hypertensive Status	Systolic BP	Diastolic BP
Diagnosis	-0.391*** (0.00862)	-2.767*** (0.490)	-3.096*** (0.295)
Age	0.0079*** (0.00116)	0.845*** (0.0708)	0.740*** (0.0442)
Age Squared	-0.000025** (0.000011)	-0.00253*** (0.00074)	-0.00744** (0.00045)
Years of Education	-0.0015 (0.0009)	-0.338*** (0.0545)	0.0074 (0.0325)
Log PCE	-0.00058 (0.0055)	-0.243 (0.336)	0.0246 (0.199)
Overweight	0.0742*** (0.00787)	4.532*** (0.478)	3.664*** (0.287)
Poor GHS	-0.00468 (0.0089)	-0.432 (0.535)	0.152 (0.317)
Male	0.01 (0.0077)	1.107** (0.466)	0.457 (0.290)
Constant	0.659*** (0.0775)	120.3*** (4.633)	72.45*** (2.768)
**Sample Size**	7585	7585	7585

Robust standard errors in parentheses.

**-significant at 5% level

***-significant at 1% level

First, we estimated the effect of a prior diagnosis on the probability of currently being hypertensive. We coded an indicator variable as 1 if the individual was considered hypertensive according to the WHO definition during the 2015 survey and 0 otherwise. We then estimated this equation using a linear probability model and reported the effects in the second column of Table 2. We also ran a probit model for robustness purposes and found almost identical results. It is easy to see that having been previously diagnosed has a significant beneficial effect on hypertensive status—individuals who are diagnosed are 39.1% less likely to be currently hypertensive, which is proof that controlling hypertension can be relatively easily achieved once a diagnose is made.

Second, we estimated the effect of a prior diagnosis on systolic and diastolic blood pressure directly. These effects are presented in columns 3 and 4 of Table 2. Once again, we see that diagnosis significantly reduces both systolic and diastolic blood pressure among hypertensive patients. Combined with the previous result, this strengthens the argument that screening and diagnosis are important and effective means of controlling high blood pressure.

Interestingly, the effects of education and per capita expenditures (which proxies for income) are mostly insignificant. Socio-economic indicators such as education and income are often cited in the literature as important determinants of health. The lack of statistical significance in our estimates does not invalidate their importance, but rather points to the main channel through which socio-economic factors affect health. At least when it comes to hypertension, it seems that education and economic well being mostly work through screening and diagnosis. Individuals with higher education and incomes are more likely to engage in screening and prevention and are therefore more likely to be diagnosed and treated for hypertension, which results in overall better health. The fact that income in particular has virtually zero direct effect on blood pressure is also most likely due to the fact that hypertension treatment is generally cheap and so once diagnosed, economic factors are not a barrier for getting treatment. As a direct policy recommendation, this implies that public spending should be mainly directed towards improving diagnosis rates. It is very likely however, that for other health conditions there might be additional direct effects of socio-economic status, especially so for health conditions where treatment is expensive. In such cases, spending on treatment, in addition to screening, might prove beneficial and even preferred.

To further investigate any potential differential impacts across demographic groups, we disaggregate the sample by gender and age and report the results of these estimations in Table 3. Anker et al. [13], for instance, note the importance of age disaggregations. For space considerations, we only report the disaggregated results for the effects on the hypertensive status, without separately looking at the effects on systolic and diastolic blood pressure.

**Table 3:** The effects of diagnosis on hypertensive status (results by gender and age).

Sub-samples	Men	Women	Young (below 40)	Old (40 and above)
**Explanatory Variables**				
Diagnosis	-0.427*** (0.0142)	-0.361*** (0.000017)	-0.647*** (0.0159)	-0.2788*** (0.0098)
Age	0.003* (0.0015)	0.015*** (0.0018)	-0.0299*** (0.0083)	0.0142*** (0.0031)
Age Squared	0.00002 (0.000015)	-0.00008*** (0.000017)	0.0006*** (0.0001)	-0.00008*** (0.00002)
Years of Education	0.0019 (0.0012)	-0.0031** (0.0014)	-0.0014 (0.0017)	-0.0007 (0.001)
Log PCE	-0.0105 (0.0074)	-0.0009 (0.0081)	-0.0081 (0.0096)	-0.0061 (0.0064)
Overweight	0.0868*** (0.0106)	0.0644*** (0.0113)	0.0726*** (0.0138)	0.069*** (0.0093)
Poor GHS	-0.0124 (0.0130)	-0.0051 (0.0120)	-0.0152 (0.0181)	-0.012 (0.0098)
Male	– (–)	– (–)	0.0096 (0.0137)	-0.0024 (0.0088)
Constant	0.928*** (0.1023)	0.4573*** (0.1140)	1.3938*** (0.1723)	0.5149*** (0.1252)
**Sample Size**	3517	4068	2318	5267

Robust standard errors in parentheses.

*-significant at 10% level

**-significant at 5% level

***-significant at 1% level

The results of these disaggregations are qualitatively in line with the full-sample results. A prior diagnosis leads to lower incidence of hypertension in both men and women, and in both young and old. A few interesting differences in magnitudes do emerge however. Men seem to benefit from a prior diagnosis slightly better than women, while the young benefit significantly more than the old. The coefficient for the below 40 years of age sub-sample is more than double in magnitude than the coefficient for the 40 and above sub-sample.

Our next efforts go towards investigating the responsible channels of disease treatment and control. As mentioned before, diagnosis itself has a simple informational role. How this information is actually used is important in understanding health patterns and eventually formulating policy. At the health care provider level, hypertension is relatively easy to get under control using a variety of prescription medication. At the individual level, hypertension can further be managed through a series of life-style changes. Patients are advised to maintain a healthy body weight, engage in moderate physical activity, reduce the intake of alcohol, saturated fat and salt, and closely monitor their blood pressure. Some of these life-style changes are costlier than others, and so we want to investigate which ones are actually followed through by diagnosed patients.

To answer these questions, we perform a series of additional regressions that estimate the effect of a prior diagnosis on a variety of variables that can lead to lower blood pressure. Table 4 presents these estimated coefficients. While not reported in the table for space considerations, all regressions include controls for respondents’ age, age squared, years of education, log of per capita expenditures (to proxy for income), poor general health status, and sex.

**Table 4:** Channels of lowering hypertension.

Dependant Variable	The Effect of a Prior Diagnosis	
	Coefficient	(Standard Error)
**Significant Channels**		
Respondent Takes Medication	0.259***	(0.00824)
Respondent Currently Smokes	-0.0432***	(0.00854)
Respondent Quit Smoking	0.105***	(0.0162)
Respondent Engages in Moderate Physical Activity	0.036***	(0.0122)
Respondent Checks Blood Pressure Regularly	0.105***	(0.00943)
**Insignificant Channels**		
Respondent Consumes Fast Food	0.00362	(0.00683)
Respondent Consumes Soft Drinks	-0.00314	(0.00876)
Respondent’s Body Mass Index	-0.0533	(0.105)
Respondent is Overweight	-0.00689	(0.0113)

Robust standard errors in parentheses.

***-significant at 1% level

The results in Table 4 show that diagnosed hypertensive patients manage their condition better than undiagnosed ones through medication, smoking less and even quitting smoking, engaging in physical activity, and monitoring their blood pressure regularly. Note that the relationship between smoking and hypertension is not settled however, in the medical literature. While most studies tend to find smoking to be a cause of hypertension, some studies contradict that. For instance, Sohn [14] finds no evidence of a linkage between smoking and being hypertensive. In spite of the lack of consensus in the literature, we do believe however, that reducing smoking is valuable in its own, even if it does not have a secondary effect to lower blood pressure.

On the other hand, there do not seem to be any major dietary changes after being diagnosed. We do not have precise data on the consumption of saturated fat or salt, but we proxy it with fast food and soft drinks consumption. Diagnosed respondents do not consume less fast food and soft drinks than undiagnosed ones, nor do they present any statistically significant differences in terms of BMI or being overweight. Note that Indonesia is a predominantly islamic country, and so alcohol consumption is not something that we can meaningfully investigate. A possible explanation for the lack of a statistically significant effect on dietary habits and BMI is the interplay between BMI and social status in developing countries. Previous studies [15] do indeed find a large wage premium associated with being overweight in Indonesia.

A direct policy implication of these results is that the public health education programs and the health care professionals who diagnose hypertensive patients should stress more the importance of a healthy diet and maintaining a healthy weight, as patients can realize additional benefits in terms of disease management through these channels. Even more so, stressing the additional benefits of all life-style factors can further enhance disease management and should be pursued through public policy. Even though diagnosed patients seem to be responsive in terms of smoking, physical activity, and monitoring their blood pressure regularly, this responsiveness seems to be pretty low overall, and much lower than the medication responsiveness. While diagnosed patients are 25.9% more likely to take medication than undiagnosed ones, they are only about 10% more likely to quit smoking and check their blood pressure regularly and only 3.6% more likely to engage in physical activity. These results are to be expected given the low cost of medication and the high cost of hard-to-make life-style changes, but they are far from ideal from a population health perspective. If we assume that all patients who are prescribed medication are also advised to adopt certain life-style changes, but some mildly hypertensive patients might only be encouraged to these changes without being prescribed medication, we would ideally like to see the magnitudes of these effects reversed.

## Conclusions

Using a large sample of hypertensive respondents from a longitudinal data set in Indonesia, we estimated the effect of a prior hypertension diagnosis on current incidence and severity of hypertension. We found that screening and diagnosis has significant positive effects and results in a lower systolic and diastolic blood pressure and in a lower overall incidence of hypertension. These results are contrary to the strand of literature that is arguing that screening and diagnosis are often ineffective and/or much more expensive than treatment. We argue that the role of diagnosis is purely informational and it is therefore essential to distinguish between different types of afflictions when performing such studies. Since different health problems have different available courses of treatment, the effects of a prior diagnose on current or future health outcomes are highly dependent on the availability and overall cost of these treatments.

We also investigated the channels through which hypertension diagnosis leads to lowering blood pressure and found medication to be the most important one. Some patients who are diagnosed are prescribed medication which then directly leads to lower blood pressure. We also found modest but significantly higher rates of certain life-style changes among diagnosed patients. Being diagnosed results in less smoking, more physical activity, and more regular monitoring of blood pressure. On the other hand, we found no significant effects on diet, BMI, and overweight status. We find these results as expected, since hypertension medication is relatively cheap, while life-style changes are often hard and costly to adopt. An interesting avenue for future research might be to consider the role of mental health and social interactions in managing hypertension. Arguably there is quite a bit of interplay between these and maintaining healthy dietary habits, smoking, and even exercising. For instance, Bustamante et al. [16] find that hypertension management, in particular through weight management, is significantly affected by the existence of social support.

That being said, given the extent of the medical literature that documents the importance of such life-style changes in managing hypertension, we argue that these should be first order items on the agenda of public policy as it relates to public health. Our overall results suggest dedicating resources both towards improving diagnosis rates and also towards education regarding the importance of adopting a healthy life-style and not only relying on medication.

As developing countries go through a health transition, from dealing mainly with infectious diseases to dealing mainly with chronic diseases, large countries like Indonesia or China have seen rapidly rising rates of hypertension among their populations. At the same time, due to the asymptomatic nature of hypertension and to a lack of resources and established medical routines, much of the afflicted population is not diagnosed and are largely unaware of being hypertensive. Given our results, it is therefore imperative for public policy in such countries to dedicate more resources towards addressing the under-diagnosis problem and educating the public on how to adopt a healthy life-style in the face of a rapidly changing economy.
